# Clinically Significant High-Grade AV Block as a Reversible Cause for Acute Kidney Injury in Hospitalized Patients—A Propensity Score Matched Cohort

**DOI:** 10.3390/jcm10112424

**Published:** 2021-05-30

**Authors:** Aviram Hochstadt, Ido Avivi, Merav Ingbir, Yacov Shacham, Ilan Merdler, Yoav Granot, Sami Viskin, Raphael Rosso, Shmuel Banai, Maayan Konigstein

**Affiliations:** 1 Division of Cardiology, Tel-Aviv Sourasky Medical Center and the Sackler School of Medicine of The Tel Aviv University, Weizman 6 St., Tel Aviv 64239, Israel; kobys@tlvmc.gov.il (Y.S.); Ilanmerdler@gmail.com (I.M.); yoavgran@gmail.com (Y.G.); samiviskin@gmail.com (S.V.); roswin1@gmail.com (R.R.); shmuelb@tlvmc.gov.il (S.B.); maayan.konigstein@gmail.com (M.K.); 2 Internal Medicine J, Tel-Aviv Sourasky Medical Center and the Sackler School of Medicine of The Tel Aviv University, Weizman 6 St., Tel Aviv 64239, Israel; idoavivi22@gmail.com (I.A.); merav2101@gmail.com (M.I.)

**Keywords:** atrio-ventricular block, acute kidney injury, pacemaker implantation, symptomatic bradycardia

## Abstract

**Background.** High-grade AV block (HGAVB) is a life-threatening condition. Acute kidney injury (AKI) which is usually caused by renal hypo-perfusion is associated with adverse outcomes. We aimed to investigate the association between AKI and HGAVB. **Methods.** This is a retrospective cohort comparing the incidence of AKI among patients with HGAVB requiring pacemaker implantation compared with propensity score matched controls. Primary outcome was the incidence of AKI at admission. Secondary outcomes were change in creatinine levels, AKI during stay, recovery from AKI, mortality and major adverse kidney events (MAKE). **Results**. In total, 80 HGAVB patients were compared to 400 controls. HGAVB patients had a higher proportion of admission AKI compared to controls (36.2% versus 21.1%, RR = 1.71 [1.21–2.41], *p* = 0.004). Creatinine changes from baseline to admission and to maximum during hospitalization, were also higher in HGAVB (*p* = 0.042 and *p* = 0.033). Recovery from AKI was more frequent among HGAVB patients (55.2% vs. 25.9%, RR = 2.13 [1.31–3.47], *p* = 0.004) with hospitalization time, MAKE and crude mortality similar (*p* > 0.158). **Conclusions.** AKI occurs in about one third of patients admitted with HGAVB, more frequent compared to controls. Patients with AKI accompanying HGAVB were likelier to recover from AKI. Further studies to explore this relationship could aid in clinical decision making for HGAVB patients.

## 1. Introduction

Clinically significant atrio-ventricular conduction block (AVB) is a relatively uncommon clinical entity, but its incidence rises with age [1]. Patients with high-grade AVB (HGAVB), defined here as type II second degree and third degree AVB, may experience abrupt decline in cardiac output (CO), increased pulmonary artery pressure along with increased right-sided filling pressures and atrio-ventricular desynchrony [2,3]. These may lead to signs and symptoms of heart failure, weakness and syncope.

Acute kidney injury (AKI), is a frequent occurrence in hospitalized patients and is associated with adverse clinical outcomes, including worsening of chronic renal failure, prolonged hospital stay, and increased mortality rates [4]. In the field of cardiology, AKI has long been associated with decompensated heart failure, with a deleterious effect on morbidity and mortality. While there are several strategies for treatment and prevention of AKI [5], it has also been shown to complicate up to a fifth of hospitalizations due to acute myocardial infarction, attributed to some of the pathophysiological mechanisms shared with AVB, including left ventricular dysfunction and low CO [6,7]. Interaction between renal function and cardiac function is long-recognized as the cardio-renal syndrome (CRS). Although AKI in the presence of HGAVB could be classified as CRS type 1, where acute decompensation of cardiac function produces AKI, current literature in both fields of cardiology and nephrology does not cite severe bradycardia and bradyarrhythmias as a specific causes of CRS [8,9]. Both decompensated heart failure and acute severe bradyarrhythmia cause decreased CO, and therefore there is a strong basis to believe that as CO decreases in HGAVB, AKI should follow.

Temporary cardiac pacing and implantation of a permanent cardiac pacemaker are standard therapies in HGAVB. These interventions, if performed shortly after hospital admission, are expected to augment CO and renal function, preventing permanent insult to the kidneys.

Data regarding the possible association between AVB and renal function is scarce; Acute and chronic kidney disease has been implicated as a cause of AVB, with serum hyperkalemia and medication overdose as suspected cause of bradyarrhythmia [10,11,12,13]. Moreover, two case reports [14,15] showed an association between AVB and acute kidney injury. However, no systematic investigation of this relationship has been previously reported. We therefore aimed to investigate the relationship between HGAVB and the occurrence of AKI in hospitalized patients.

## 2. Materials and Methods

To estimate the incidence of AKI during HGAVB, our study population included all patients admitted to the Tel-Aviv Sourasky Medical center between 2007 and 2020, with diagnosis of HGAVB requiring pacemaker implantation, whose baseline renal function was known. All data were obtained using the center’s computerized medical record system which registers diagnoses, chronic and administered medications, results of clinical, imaging and laboratory tests performed, and procedures done during and after admission. This study was approved by the institution’s internal review board for clinical trials according to the declaration of Helsinki and its amendments.

### 2.1. Baseline Renal Function

We defined patients’ baseline creatinine as the lowest recorded creatinine measured in a biochemistry assay at our center’s laboratory within 1 year and up to 7 days prior to their index admission. This time period was selected to prevent underestimation of baseline creatinine due to expected deterioration in renal function over time, and possible overestimation by a measurement too close to the index event. Patients’ blood test from community laboratories are not recorded in our institution’s database and did not influence study definitions, this also prevented errors due to different calibrations of creatinine measurements between laboratories. Baseline estimated glomerular filtration rate (eGFR) was computed using the CKD-EPI Formula [16].

### 2.2. Study Population

To compare the incidence of AKI we compared patients with clinically significant HGAVB to controls hospitalized in the cardiology department with other cardiologic pathologies without AVB.

HGAVB was defined as admission to hospital with one of the following diagnoses: complete AV block, second degree Mobitz type II AV block, undifferentiated 2:1 AV block and third degree AVB. Clinically significant AVB was defined as HGAVB that required pacemaker implantation during the index hospitalization.

The control group consisted of patients without HGAVB who were admitted non-electively to the cardiology division. Common diagnoses of patients admitted to the cardiology division include non-ST elevation acute coronary syndrome, decompensated heart failure, myocarditis, suspected malignant syncope, and arrhythmias for investigation. We excluded patients admitted to the cardiology intensive care unit (ICU), as these patients have a high proportion of diagnoses of ST elevation myocardial infarction (STEMI) and cardiogenic shock, which are established causes of AKI [6,7].

### 2.3. Outcomes

AKI was defined according to the definition of the Kidney Disease: Improving Global Outcomes (KDIGO) Practice Guideline for Acute Kidney Injury, as either increase in serum creatinine by ≥0.3 mg/dL, or increase in serum creatinine to ≥1.5 times from baseline [17].

The primary outcome was the incidence of AKI at admission (using first available creatinine level taken within minutes to hours from arrival at the emergency department) compared with baseline creatinine level. This was chosen as it is relatively insensitive to length of hospital stay and given treatment while admitted.

Secondary outcomes included the difference in creatinine level between baseline and admission, AKI during hospitalization (defined as elevation of serum creatinine between established baseline and maximally recorded creatinine), recovery from AKI (defined as return to <0.3 and <1.5 times of baseline creatinine before discharge), hospitalization time, need for renal replacement therapy (RRT), all-cause mortality and post hospital major adverse kidney event (MAKE, defined as RRT, decrease in GFR to more than 25% than baseline or mortality).

### 2.4. Statistical Methods

Data compliance with normality assumptions was assessed using histograms and QQ-plots. All data for continuous variables is presented as either mean (±SD) or median (IQR), as appropriate, and, for categorical variables as *n* (%). Hazard Ratios (HRs), Odds Ratios and relative risk (RR) are presented as HR, OR or RR with (95% CI).

As the groups varied significantly in baseline characteristics (Supplementary Table S1), they were matched in a 1:5 ratio to obtain balanced groups preserving statistical power. Matching was done using a greedy nearest neighbor matching technique to reduce the propensity score distance as much as possible. Assessment of balance was done using standardized mean differences (SMD).

Continuous variables were compared using a Mann–Whitney U test or a Welsh t-test, as appropriate. Categorical variables were compared using Chi-square. Kaplan–Meier curves were used to assess differences in mortality during follow-up amongst different groups. The significance of the difference between individual curves was assessed using a Log-rank test. Cox proportional hazards models were used to compute HRs. The proportional hazard assumption was confirmed using Schoenfeld residuals plots. Multivariate logistic regressions were used to evaluate outcomes independent of other covariates. To create the multivariate model, a combined backward and forward Akaike’s Information Criterion (AIC) dependent stepwise approach was used to select the covariates in the final models using a seed model consisting of all relevant variables. To prevent omission of data in the regression, missing values of variables were imputed using a random forest method. A two-tailed *p*-value of less than 0.05 was considered statistically significant. All statistical analyses were performed using R version 3.6.3 (R Foundation for Statistical Computing, Vienna, Austria).

## 3. Results

**Figure 1:**
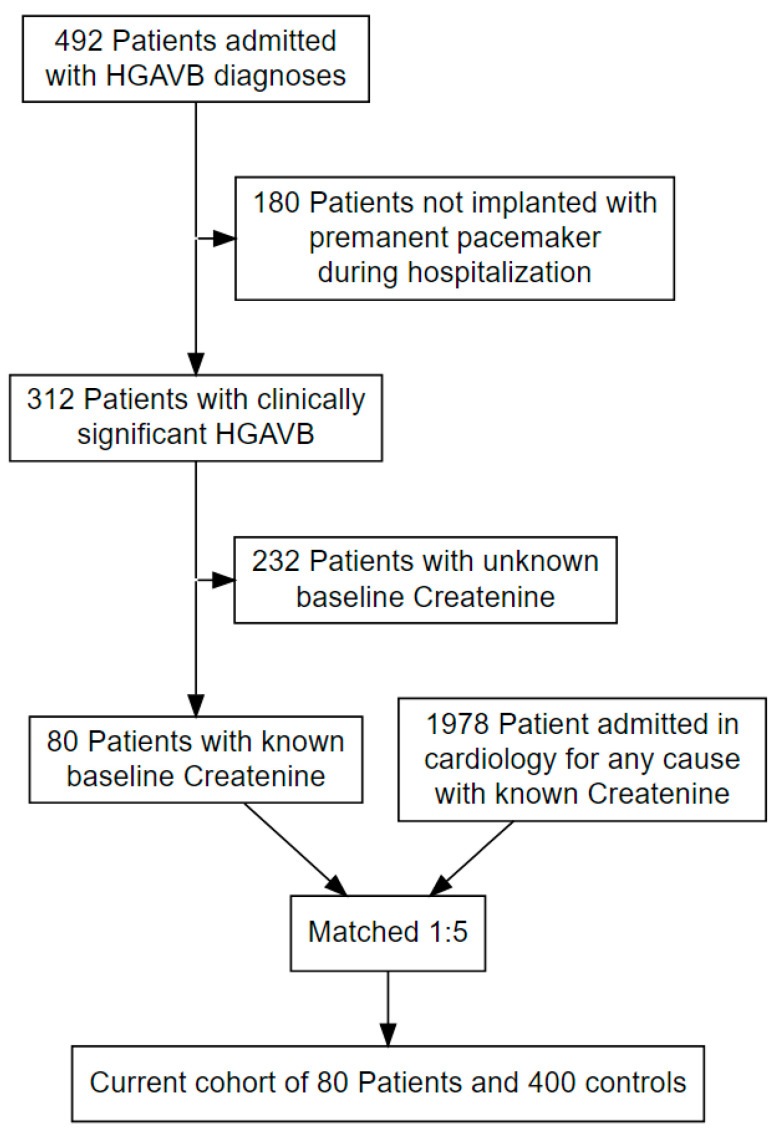
Flow chart describing inclusion of patients to the cohort.

Out of 492 patients admitted with HGAVB, the final study group consisted of 80 patients with clinically significant HGAVB and known baseline creatinine levels. These patients were matched using a propensity score matching technique to create two balanced groups in a 1:5 ratio (a total of 80 patients in the HGAVB and 400 patients in the control group, Figure 1). Mean age was 79.5 ± 10.3 years and about half (229 (47.7%)) were females. Baseline eGFR was 66.9 ± 21.0 mL/min/1.73 m^2^. Patients from both groups had a baseline creatinine tested at a median of 192 (82–286) days prior to their admission with no significant difference between groups (SMD = 0.056, *p* = 0.672).

Other baseline characteristics and balance measures are summarized in Table 1. Patients with HGAVB had a lower heart rate (66.0 ± 19.3 vs. 75.3 ± 20.6, *p* = 0.001) yet similar values of systolic and diastolic blood pressure measurements (*p* > 0.718). Other clinical characteristics at hospital admission are detailed in Table 2.

### 3.1. Primary Outcome

**Figure 2:**
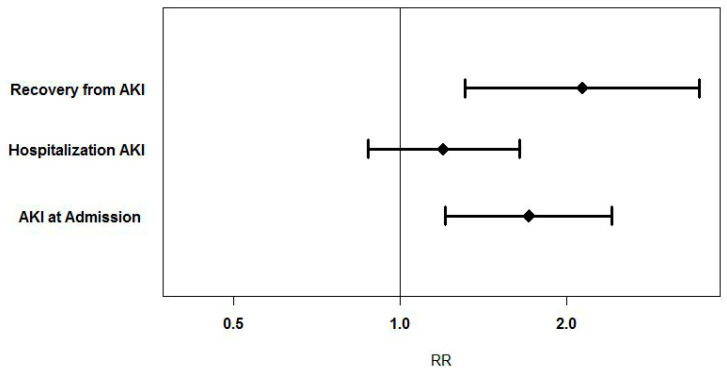
Relative Risk of the various outcomes described, with 95% CIs.

The primary outcome, incidence of AKI on admission, occurred in 29 (36.2%) patients in the HGAVB group and 85 (21.2%) in the control group (RR = 1.71 [1.21–2.41], *p* = 0.004, Table 3, Figure 2).

### 3.2. Secondary Outcomes

**Figure 3:**
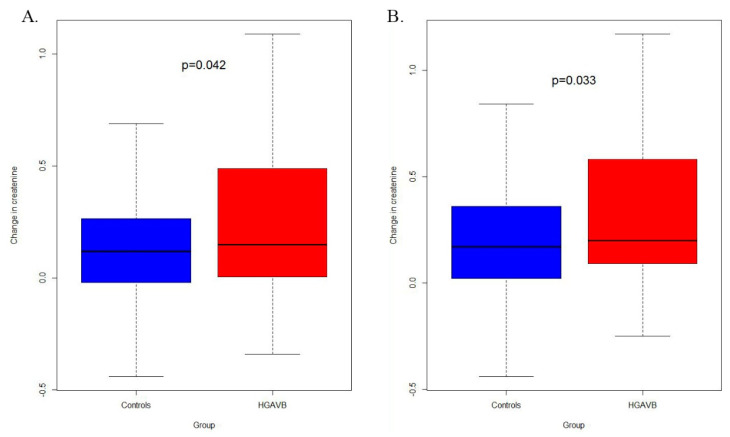
Box plots of change in creatinine level in mg/dL between groups of HGAVB and controls between (**A**) baseline creatinine and admission creatinine, and (**B**) baseline creatinine and maximum level of creatinine during hospitalization.

Differences between creatinine levels at baseline and admission, and between baseline and maximum creatinine level during hospitalization, were higher in the HGAVB group compared to controls (*p* < 0.041 for both, Table 3 and Figure 3), yet there was no significant difference in the frequency of AKI during hospitalization (between baseline and maximum creatinine level measured during admission) (*p* = 0.279, Table 3, Figure 2).

Recovery from AKI was significantly more common in the HGAVB group (RR 2.13 [1.31–3.47], *p* = 0.004, Table 3, Figure 2), showing that more AKI patients returned to their baseline creatinine level in the HGAVB group compared to controls.

**Figure 4:**
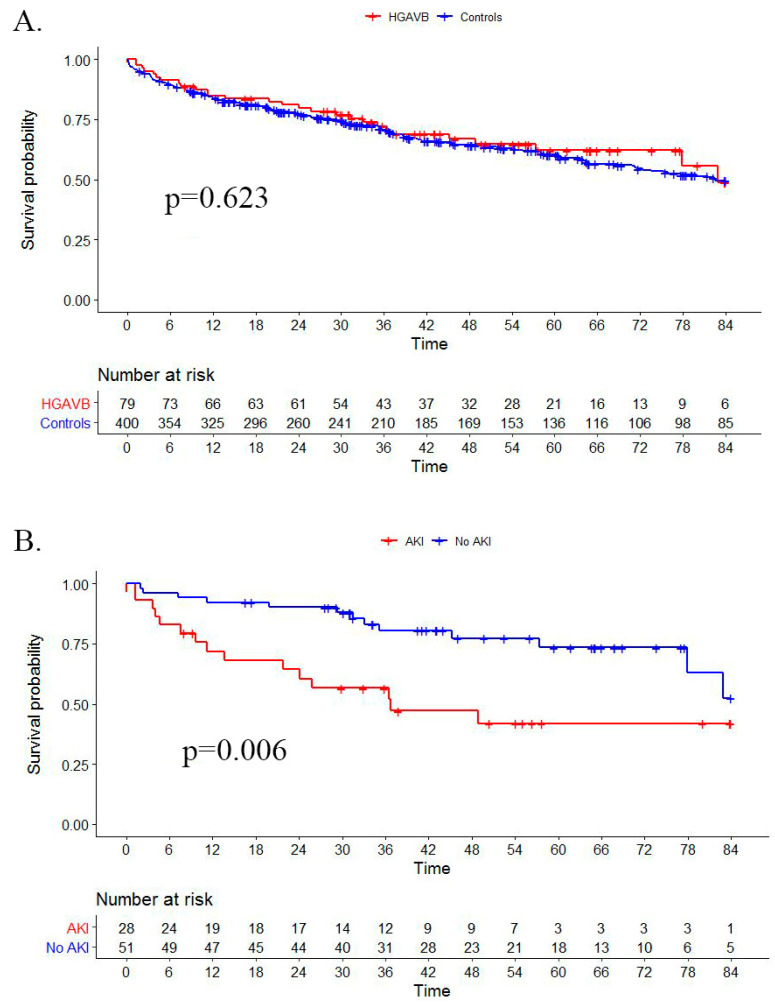
Kaplan–Meier curves of overall mortality among (**A**) patients with HGAVB compared with controls and (**B**) patient with HGAVB with AKI compared to those without AKI.

Hospitalization time, future need for dialysis, MAKE and long-term mortality were similar between groups (*p* > 0.346 for all comparisons, Table 3, Figure 4A). Long-term mortality was higher in patients with AKI compared to patients without AKI in both groups (HR 2.59 (1.77–3.78), *p* < 0.001, and HR of 2.76 (1.31–5.82) (*p* = 0.006) for patients without and with HGAVB, respectively, Figure 4B), with no significant interaction between AKI and HGAVB status (*p* = 0.720 for interaction).

### 3.3. Predictors of AKI in Patients with HGAVB

Among all measured parameters in the presence of HGAVB, AKI was independently and significantly associated with higher leukocyte count, higher heart rate, use of Renin-Angiotensin-Aldosterone system (RAAS) inhibitors, lower platelet counts and lower baseline eGFR. Use of beta blockers and higher blood pressure seemed to be protective against AKI, albeit reaching only borderline significance (Table 4).

## 4. Discussion

Acute kidney injury (AKI) is common among hospitalized patients and has been associated with numerus cardiac and non-cardiac factors. Kidney injury caused directly and indirectly by myocardial infarction and heart failure has been previously described [5,6], and the cardio-renal syndrome has been extensively researched and described albeit not in the context of severe bradyarrhythmia [8,9]. In the present study, we aimed to investigate, for the first time, the relationship between HGAVB and AKI. AKI secondary to bradyarrhythmias has been previously described only in two case reports-one case in which complete AVB manifested as AKI amongst other various complications [14] and one case with sinus bradycardia and intermittent complete AV block which manifested as AKI [15]. In both cases, kidney injury improved after the bradycardia was resolved following cardiac pacemaker implantation. Here we report, that in a cohort of patients admitted to hospital because of HGAVB requiring pacemaker implantation, more than one third of patients developed AKI, and that this proportion is significantly higher compared to a matched group of patients admitted non-electively for other cardiac reasons. This proportion is comparable to the 5%–30% rates described in patients with STEMI [6,18,19] and the 32%–40% described in patients with heart failure and cardio-renal syndrome [5].

We also show that AKI is associated with higher long-term mortality with an HR of 2.3 (1.6–3.2) for the entire study population and an HR of 2.8 [1.3–5.8] specifically among patients with clinically significant HGAVB. These findings are concordant with previously reported data regarding the impact of AKI on hospital mortality [20,21,22].

Importantly, we report significantly higher fraction of patients who experienced resolution of their AKI in the HGAVB group. This finding may be explained by the fact that HGAVB has a relatively rapid and effective therapeutic solution in the form of temporary pacing and permanent pacemaker implantation and may indicate that most patients arriving to the hospital with HGAVB and AKI are in a relatively early and reversible state of their kidney injury.

Potential mechanisms for the impact of HGAVB on renal function start from the obvious–the direct hemodynamic effects of HGAVB on circulation [2,3]: as heart rate decreases, the cardiovascular system loses its ability to control CO by tachycardia and can only compensate by increasing stroke volume which is limited, especially in patients with previous cardiovascular diseases [23]. The decrease in CO can, by itself, reduce glomerular filtration rate, as one is derived from the other. The cardiovascular regulatory mechanisms can also cause renal injury-as one compensatory mechanism is vasoconstriction which can affect the renal vasculature and decrease renal blood flow. Another hemodynamic change that may occur as a result of CO decreasing, is increased systemic congestion which causes high central venous pressure, directly affecting renal vein and kidney perfusion pressure and also resulting in increased interstitial pressure with tubular collapse and further decline in the kidney function. Another suggested explanation is neurohormonal changes during HGAVB, such as activation of the RAAS which closely affects renal circulation by direct glomerular vasoactivity [24]. Furthermore, Angiotensin II activates NADPH oxidase in both the heart and kidneys which increases production of reactive oxygen species leading to reduced nitric oxide bioavailability and vasoconstriction [25]. This explanation is supported by the very high OR (8.0 (1.7–48.0)) in the use of RAAS inhibitors in HGAVB patients with AKI.

As the mechanisms of AKI in HGAVB are at least partially reversible, pacemaker implantation could alleviate not only the symptoms of AVB but also its renal manifestations. Hence, considering the impact of AKI on mortality, according to the results of this study, it may be reasonable to treat HGAVB patients in expeditious manner, either medically (using chronotropic medications, careful hydration and withholding RAAS inhibitors) or invasively (by temporary or permanent pacing) in order to prevent permanent damage. This approach needs to be further evaluated in randomized controlled trials.

**Limitations.** This is a small, single center, retrospective study and should not be extrapolated outside its context. Studies with larger sample sizes and reproducibility in multiple centers are needed to enhance external validity. Diagnoses were acquired through computerized patients’ records which may be susceptible to errors, yet, as we only selected patients who had a pacemaker implanted during their stay (a coding which is very reliable due to its use for compensation from insurers), error should be diminished to a minimum.

Our method of acquiring the baseline creatinine has a few possible flaws. It is susceptible both to overestimation, because tests were taken during admission to the hospital (or the emergency department) where AKI can manifest for other causes, and on the other hand susceptible to underestimation inherent to taking the minimal value out of several measurements. Nevertheless, as this is the method we use in clinical practice to estimate baseline laboratory measurements, and because any biases should be similar in cases and in controls, specifically due to matching according also to the time period the test was taken before admission, we believe any inaccuracies should not affect the final results.

Finally, there is a concern that the definition of AKI used (creatinine change ≥ 0.3 mg/dL or ≥1.5 times higher than baseline) may be too low, as this definition was originally meant to be used for patients during their hospitalization who arise suspicion of kidney function deterioration [17]. Yet, this cutoff offers a sense of clinically significant creatinine elevation. Moreover, AKI according to this definition was associated with adverse clinical outcomes in numerous studies [20,22].

Despite the above limitations regarding the external and internal validity of this research, the conclusion is sound and calls for other, prospective trials, to examine the relationship between bradyarrhythmias and renal manifestations, possibly elaborating on the mechanisms involved. The practical utility of this and future studies might change the approach to HGAVB and renal failure, such as a recommendation to expedite pacemaker implantation (<24 h) in cases of HGAVB with impaired renal function, consideration of hydration upon admission, and careful monitoring of renal function during and following hospital stay.

## 5. Conclusions

In conclusion, patients with clinically significant HGAVB presented higher rates of acute kidney injury compared to controls, along with better recovery of renal function following treatment.

## Figures and Tables

**Table 1:** Baseline characteristics of the entire cohort, high grade AV block (HGAVB) patients and controls.

Parameter	Overall	No HGAVB	HGAVB	*p*-Value	SMD
n	480	400	80		
Age (years)—Mean ± SD	79.5 ± 10.3	79.3 ± 10.2	80.4 ± 10.8	0.383	0.105
Female Gender (%)	229 (47.7)	189 (47.2)	40 (50.0)	0.653	0.055
History of hypertension (%)	261 (54.4)	215 (53.8)	46 (57.5)	0.539	0.076
History of dyslipidemia (%)	191 (39.8)	156 (39.0)	35 (43.8)	0.428	0.097
History of diabetes (%)	128 (26.7)	104 (26.0)	24 (30.0)	0.460	0.089
History of IHD (%)	144 (30.0)	121 (30.2)	23 (28.7)	0.789	0.033
History of heart failure (%)	55 (11.5)	45 (11.2)	10 (12.5)	0.749	0.039
Beta blocker treatment (%)	187 (39.0)	154 (38.5)	33 (41.2)	0.645	0.056
ACEI or ARB treatment (%)	194 (40.4)	160 (40.0)	34 (42.5)	0.677	0.051
MRA treatment (%)	16 (3.3)	13 (3.2)	3 (3.8)	0.820	0.027
Baseline eGFR (mL/kg/min/1.73 m^2^)—Mean ± SD	66.9 ± 21.0	66.8 ± 21.3	67.0 ± 19.8	0.963	0.006
Baseline creatinine (mg/dL)—Median [IQR]	0.9 (0.8, 1.1)	0.9 (0.8, 1.2)	0.9 (0.7, 1.1)	0.417	0.010
Baseline creatinine acquiring prior to admission (days)—Median [IQR]	191.7 (82.3, 286.3)	191.7 (79.5, 288.2)	192.8 (94.6, 283.3)	0.672	0.056

IHD—Ischemic Heart Disease; ACEI—Angiotensin Converting Enzyme Inhibitor; ARB—Angiotensin Receptor Blockers; MRA—Mineralocorticoid Receptor Antagonist; eGFR—estimated Glomerular Filtration Rate.

**Table 2:** Clinical characteristics of patients with and without high grade AV block (HGAVB).

	No HGAVB	HGAVB	*p*-Value
n			
Heart Rate (bpm)—Mean ± SD	75.2 ± 20.3	66.0 ± 19.4	0.001
Systolic Blood Pressure (mmHg)—Mean ± SD	137.0 ± 24.1	142.3 ± 21.9	0.185
Diastolic Blood Pressure (mmHg)—Mean ± SD	72.5 ± 14.3	75.2 ± 15.6	0.273
Oxygen Saturation (%)—Mean ± SD	95.7 ± 3.8	95.7 ± 3.8	1.000
Hemoglobin (g/dL)—Mean ± SD	12.2 ± 1.8	12.1 ± 1.8	0.665
WBC (cells/nL)—Mean ± SD	8.9 ± 4.8	13.3 ± 37.5	0.025
PLT (cells/nL)—Mean ± SD	226.5 ± 96.2	208.9 ± 68.3	0.123
Serum Na (mmol/L)- Mean ± SD	138.2 ± 3.8	137.4 ± 4.3	0.113
Serum K (mmol/L)—Mean ± SD	4.2 ± 0.5	4.4 ± 0.6	0.021
Blood Albumin (g/dL)—Mean ± SD	38.4 ± 4.4	37.9 ± 4.2	0.403

bpm—beats per minute; WBC—white blood cells, PLT—platelets.

**Table 3:** Primary and secondary endpoints of patients with and without high grade AV block (HGAVB).

	No HGAVB	HGAVB	*p*-Value
AKI on admission (%)	85 (21.2)	29 (36.2)	0.004
AKI during hospitalization (%)	113 (33.4)	30 (40.0)	0.279
Recovery from AKI during stay	22 (25.9)	16 (55.2)	0.004
Admission creatinine change from baseline (mg/dL)—median [IQR]	0.12 (−0.02, 0.26)	0.15 (0.01, 0.49)	0.042
Maximum creatinine change from baseline (mg/dL)—median [IQR]	0.17 (0.02, 0.36)	0.20 (0.09, 0.58)	0.033
Future RRT (%)	17 (4.2)	4 (5.0)	0.765
MAKE (%)	271 (67.7)	46 (58.0)	0.861
Mortality (%)	202 (50.4)	41 (51.2)	0.623
Length of hospitalization (Days)—median [IQR]	3.19 (1.98, 5.40)	2.71 (1.88, 5.00)	0.346

AKI—Acute Kidney Injury; RRT—renal replacement therapy. MAKE—Major adverse kidney events.

**Table 4:** Results of multivariate logistic regression for the effect of various covariates on acute kidney injury incidence in patients with high grade AV block.

	OR [95% CI]	*p*-Value
Blood WBC count (per cell/nL)	1.46 (1.14–1.98)	0.005
Heart rate (per bpm)	1.05 (1.01–1.09)	0.012
Use of ACEI or ARBs or MRAs	8.04 (1.73–47.96)	0.013
Blood Platelet count (per cell/nL)	0.99 (0.97–1)	0.015
Baseline eGFR (per mL/h/m^2^)	0.96 (0.92–0.99)	0.017
Use of Beta Blockers	0.14 (0.02–0.66)	0.019
Systolic blood pressure (per mmHg)	0.96 (0.92–1)	0.067
History of hypertension	3.3 (0.68–18.5)	0.147

OR—Odds Ratio; WBC—White Blood Cells; ACEI—Angiotensin Converting Enzyme Inhibitor; ARB—Angiotensin Receptor Blockers; MRA—Mineralocorticoid Receptor Antagonist; eGFR—estimated Glomerular Filtration Rate.

## Data Availability

Data will be available to researchers upon approval of the Tel-Aviv medical center Institutional Review Board.

## Supplementary Materials

The following are available online at https://www.mdpi.com/article/10.3390/jcm10112424/s1, Table S1: Baseline characteristics of the entire cohort before matching., Table S2: Seed model for multivariate logistic regression shown in Table 4.
