# Supplementary material for: Clinically Significant High-Grade AV Block as a Reversible Cause for Acute Kidney Injury in Hospitalized Patients—A Propensity Score Matched Cohort
Source: J Clin Med. 2021 May 30;10(11):2424. doi: 10.3390/jcm10112424 (PMC8199146; doi:10.3390/jcm10112424)
Supplement: Supplementary file 1 [file jcm-10-02424-s001.zip › jcm-1238272-supplementary.pdf]

Supplementary Table

Table S1. Baseline characteristics of the entire cohort before matching.

Parameter	No HDAVB	HDAVB	p-value	SMD
n	1978	81		
Age (years) - Mean ± SD	68.8 (15.5)	80.3 (10.9)	<0.001	0.862
Female Gender (%)	696 (35.2)	41 (50.6)	0.005	0.316
History of hypertension (%)	795 (40.2)	46 (56.8)	0.003	0.337
History of dyslipidemia (%)	638 (32.3)	35 (43.2)	0.039	0.227
History of diabetes (%)	490 (24.8)	24 (29.6)	0.322	0.109
History of ischemic heart disease (%)	1013 (51.2)	24 (29.6)	<0.001	0.451
History of heart failure (%)	454 (23.0)	10 (12.3)	0.025	0.281
Beta blocker treatment (%)	694 (35.1)	33 (40.7)	0.297	0.117
ACEI or ARB treatment (%)	696 (35.2)	34 (42.0)	0.211	0.140
Mineralocorticoid agonist treatment (%)	144 (7.3)	3 (3.7)	0.220	0.157
Baseline eGFR (ml/kg/min/1.73m²) - Mean ± SD	68.6 (24.3)	67.2 (19.8)	0.618	0.062
Baseline creatinine (mg/dl) - Median [IQR]	1.0 [0.8, 1.2]	0.9 [0.7, 1.1]	0.001	0.174
Baseline creatinine acquiring prior to admission (days) - Median [IQR]	128.1 [49.9, 243.1]	189.8 [90.0, 282.4]	0.001	0.365

High grade AV block (HGAVB); IHD- Ischemic Heart Disease; ACEI – Angiotensin Converting Enzyme Inhibitor; ARB Angiotensin Receptor Blockers; MRA Mineralocorticoid Receptor Antagonist; eGFR estimated Glomerular Filtration Rate.

Table S2. Seed model for multivariate logistic regression shown in Table-4.

Parameter	OR [95% CI]	p-value
Age (years)	0.99 [0.9 - 0.818]	0.818
Baseline Creatinine (mg/dL)	1.28 [0.03 - 0.911]	0.911
Diastolic Blood Pressure (mmHg)	1.01 [0.93 - 0.789]	0.789
Saturation (%)	0.8 [0.56 - 0.202]	0.202
Pulse (bpm)	1.05 [0.99 - 0.108]	0.108
Systolic Blood Pressure (mmHg)	0.94 [0.87 - 0.112]	0.112
Hemoglobin (g/dL)	0.73 [0.39 - 0.276]	0.276
WBC (cells/nL)	1.55 [1.15 - 0.009]	0.009
PLT (cells/nL)	0.98 [0.97 - 0.099]	0.099
Na (cells/nL)	1.11 [0.92 - 0.344]	0.344
K (cells/nL)	1.02 [0.2 - 0.982]	0.982
eGFR (ml/min/m²)	0.96 [0.87 - 0.492]	0.492
History of hypertension	3.22 [0.42 - 0.269]	0.269
History of hyperlipidemia	1.2 [0.14 - 0.869]	0.869
History of Diabetes	0.94 [0.1 - 0.95]	0.95
Ischemic Hear disease	1.86 [0.23 - 0.558]	0.558
Heart Failure	2.23 [0.08 - 0.637]	0.637
Use of beta blockers	0.12 [0.01 - 0.049]	0.049
Use of ACE/ARB/MRA	5.02 [0.57 - 0.169]	0.169
Female Gender	1.15 [0.15 - 0.894]	0.894

OR – Odds Ratio; WBC- White Blood Cells; ACEI – Angiotensin Converting Enzyme Inhibitor; ARB Angiotensin Receptor Blockers; MRA Mineralocorticoid Receptor Antagonist; eGFR estimated Glomerular Filtration Rate.
